# The complete genome sequence of *Corynebacterium pseudotuberculosis *FRC41 isolated from a 12-year-old girl with necrotizing lymphadenitis reveals insights into gene-regulatory networks contributing to virulence

**DOI:** 10.1186/1471-2164-11-728

**Published:** 2010-12-30

**Authors:** Eva Trost, Lisa Ott, Jessica Schneider, Jasmin Schröder, Sebastian Jaenicke, Alexander Goesmann, Peter Husemann, Jens Stoye, Fernanda Alves Dorella, Flavia Souza Rocha, Siomar de Castro Soares, Vívian D'Afonseca, Anderson Miyoshi, Jeronimo Ruiz, Artur Silva, Vasco Azevedo, Andreas Burkovski, Nicole Guiso, Olivier F Join-Lambert, Samer Kayal, Andreas Tauch

**Affiliations:** 1 Institut für Genomforschung und Systembiologie, Centrum für Biotechnologie, Universität Bielefeld, Universitätsstraße 27, D-33615 Bielefeld, Germany; 2 CLIB Graduate Cluster Industrial Biotechnology, Centrum für Biotechnologie, Universität Bielefeld, Universitätsstraße 27, D-33615 Bielefeld, Germany; 3 Lehrstuhl für Mikrobiologie, Friedrich-Alexander-Universität Erlangen-Nürnberg, Staudtstraße 5, D-91058 Erlangen, Germany; 4 Bioinformatics Resource Facility, Centrum für Biotechnologie, Universität Bielefeld, Universitätsstraße 27, D-33615 Bielefeld, Germany; 5 AG Genominformatik, Technische Fakultät, Universität Bielefeld, Universitätsstraße 25, D-33615 Bielefeld, Germany; 6 Laboratório de Genética Celular e Molecular, Departamento de Biologia Geral, Instituto de Ciências Biológicas, Universidade Federal de Minas Gerais, Av. Antonio Carlos 6627, Pampulha, Belo Horizonte, MG, Brazil; 7 Cellular and Molecular Parasitology Laboratory, Rene Rachou Research Center, Oswaldo Cruz Foundation (FIOCRUZ), Belo Horizonte, MG, Brazil; 8 Instituto de Ciências Biológicas, Universidade Federal do Pará, Rua Augusto Corrêa, 01 - Guamá, Belém, PA, Brazil; 9 Institut Pasteur, Unité de Prévention et Thérapies Moléculaires des Maladies Humaines, National Centre of Reference of Toxigenic Corynebacteria, 25 rue du Dr. Roux, 75724 Paris Cedex 15, France; 10 Medical Faculty, University Paris Descartes, Hospital Necker Enfants Malades, Department of Microbiology, 147 rue de Sèvres, 75015 Paris, France; 11 Medical Faculty, University Rennes 1, Hospital Pontchaillou, Department of Microbiology, 2 rue Henri le Guilloux, 35000 Rennes, France

## Abstract

**Background:**

*Corynebacterium pseudotuberculosis *is generally regarded as an important animal pathogen that rarely infects humans. Clinical strains are occasionally recovered from human cases of lymphadenitis, such as *C. pseudotuberculosis *FRC41 that was isolated from the inguinal lymph node of a 12-year-old girl with necrotizing lymphadenitis. To detect potential virulence factors and corresponding gene-regulatory networks in this human isolate, the genome sequence of *C. pseudotuberculosis *FCR41 was determined by pyrosequencing and functionally annotated.

**Results:**

Sequencing and assembly of the *C. pseudotuberculosis *FRC41 genome yielded a circular chromosome with a size of 2,337,913 bp and a mean G+C content of 52.2%. Specific gene sets associated with iron and zinc homeostasis were detected among the 2,110 predicted protein-coding regions and integrated into a gene-regulatory network that is linked with both the central metabolism and the oxidative stress response of FRC41. Two gene clusters encode proteins involved in the sortase-mediated polymerization of adhesive pili that can probably mediate the adherence to host tissue to facilitate additional ligand-receptor interactions and the delivery of virulence factors. The prominent virulence factors phospholipase D (Pld) and corynebacterial protease CP40 are encoded in the genome of this human isolate. The genome annotation revealed additional serine proteases, neuraminidase H, nitric oxide reductase, an invasion-associated protein, and acyl-CoA carboxylase subunits involved in mycolic acid biosynthesis as potential virulence factors. The cAMP-sensing transcription regulator GlxR plays a key role in controlling the expression of several genes contributing to virulence.

**Conclusion:**

The functional data deduced from the genome sequencing and the extended knowledge of virulence factors indicate that the human isolate *C. pseudotuberculosis *FRC41 is equipped with a distinct gene set promoting its survival under unfavorable environmental conditions encountered in the mammalian host.

## Background

*Corynebacterium pseudotuberculosis *is generally regarded as an important animal pathogen and the etiological agent of a disease that is commonly called caseous lymphadenitis [1,2]. This bacterium is predominantly isolated from sheep and goats (biovar *ovis*), but has been recognized also in other animals, including horses and cattle (biovar *equi*) [1]. The importance of caseous lymphadenitis varies greatly around the world, but this disease is found in all major sheep and goat production areas [2]. *C. pseudotuberculosis *is a significant cause of morbidity in sheep and goats, and caseous lymphadenitis in these animals resulted in economic losses, for instance in wool, milk and meat production [2,3]. *C. pseudotuberculosis *is a facultative intracellular pathogen that is able to survive and grow in macrophages, thus escaping the immune response of the host [1,4]. A close phylogenetic relationship between *C. pseudotuberculosis *and *Corynebacterium ulcerans *was proposed as both species are unique among the corynebacteria in producing phospholipase D. The sphingomyelin-degrading enzyme is regarded as the major virulence factor for *C. pseudotuberculosis *[5,6]. This exotoxin promotes the hydrolysis of ester bonds in sphingomyelin in mammalian cell membranes and contributes to the spread of the bacterium from the initial site of infection to secondary sites within the host.

Although infections due to *C. pseudotuberculosis *are predominantly observed in sheep and goats, infections due to this pathogen also occur in humans [7,8]. The number of human infections is rare, but it might be underestimated as corynebacteria are often considered as skin contaminants in clinical specimens [9]. Published cases of human infections by *C. pseudotuberculosis *usually presented as suppurative lymphadenitis [7,8], with the exception of one case of eosinophilic pneumonia [10]. Most patients revealed a classical risk exposure of close contact with animals, in particular with sheep. *C. pseudotuberculosis *infects humans via superficial wounds, forming abscesses in the regional draining lymph nodes after an incubation period ranging from weeks to months. In most of the published cases, antibiotic treatment alone was unsuccessful and the antimicrobial therapy was therefore supplemented by surgical interventions [7,8]. The general problem in achieving an effective treatment of *C. pseudotuberculosis *infections in humans and animals is probably related to the facultative intracellular lifestyle of this bacterium, as it can survive and multiply in macrophages [4]. The cell death and subsequent release of the pathogen lead to necrotic lesions and the formation of thick collagen capsules that cannot be penetrated by antibiotics [11].

In the present study, we characterize the genome of *C. pseudotuberculosis *FRC41 that was isolated from the inguinal lymph node of a 12-year-old French girl with necrotizing lymphadenitis [12]. This patient had no underlying disease or predisposing conditions. The diagnosis of necrotizing lymphadenitis was supported by 16 S rDNA gene sequencing, a taxonomic classification of the bacterial isolate and Western blot assays revealing the presence of phospholipase D in the patient's serum. The patient relapsed many times despite a surgical drainage and an adapted antimicrobial treatment, although *C. pseudotuberculosis *FRC41 turned out to be highly susceptible to a wide range of antibiotics *in vitro*. However, the patient recovered after a broad spectrum intravenous antimicrobial therapy with imipenem-cilastatin, rifampin and ofloxacin for four months, followed by an oral therapy with rifampin and ofloxacin for six months [12]. Here, we report the functional annotation of the complete genome sequence of *C. pseudotuberculosis *FRC41, the detection of potential virulence factors and the deduced gene-regulatory networks controlling their expression.

## Results and discussion

### Pyrosequencing and annotation of the *C. pseudotuberculosis *FRC41 genome

**Figure 1:**
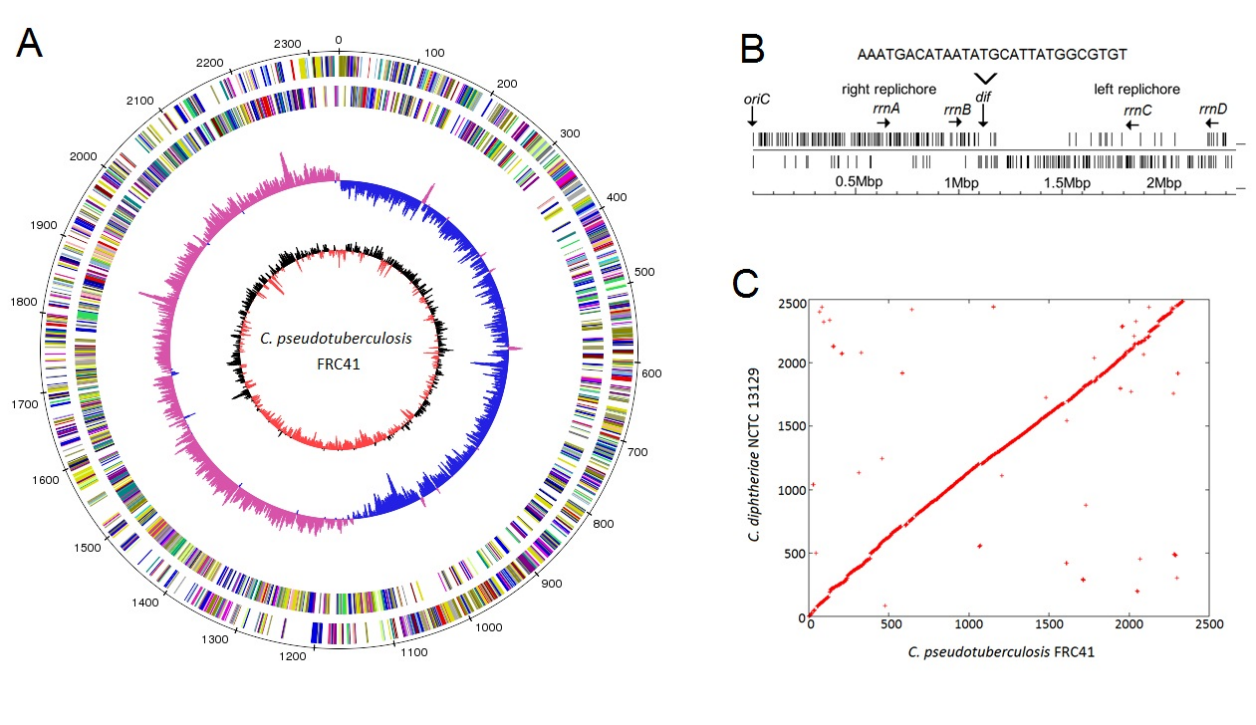
**Annotation and analysis of the *C. pseudotuberculosis *FRC41 genome sequence**. **(A)**, Plot of the annotated *C. pseudotuberculosis *FRC41 chromosome. The circles represent from the outside: circle 1, DNA base position [kb]; circle 2, protein-coding regions transcribed clockwise; circle 3, protein-coding regions transcribed anticlockwise; circle 4, G/C skew plotted using a 10-kb window; circle 5, G+C content plotted using a 10-kb window. The protein-coding regions are coloured according to their functional classification into the Clusters of Orthologous Groups of proteins [111]. **(B)**, Architecture imparting sequences in the *C. pseudotuberculosis *FRC41 chromosome. The distribution of the octamers G(A/T/C)GGGGGA and (T/C)GGGGGAG on the leading and lagging strands is shown. The origin of replication (*oriC*) is marked. The deduced *dif *locus is located at around 1.1 Mbp of the chromosomal map. The 28-bp sequence of the predicted *dif *region is shown. The location of the four rRNA operons (*rrnA*-*rrnD*) on the leading strands is indicated. **(C)**, Synteny between the chromosomes of *C. pseudotuberculosis *FRC41 and *C. diphtheriae *NCTC 13129. The X-Y plot is composed of dots forming syntenic regions between both chromosomes. The dots represent predicted *C. pseudotuberculosis *FRC41 proteins having an orthologue in the genome of *C. diphtheriae *NCTC 13129 with co-ordinates corresponding to the position of the respective coding region in each genome sequence and indicated in kb. Orthologous proteins were detected by reciprocal best BLASTP matches.

The DNA sequence of the *C. pseudotuberculosis *FRC41 chromosome was determined by a whole-genome shotgun approach using pyrosequencing. A quarter of a sequencing run with the Genome Sequencer FLX Instrument yielded 286,938 reads and 94,447,635 bases that were assembled into ten large contigs (≥ 500 bases) and one small contig (313 bases), indicating a very low number of repetitive sequences in the *C. pseudotuberculosis *FRC41 genome. A search for repetitive DNA elements in the complete genome sequence revealed the absence of insertion sequences in *C. pseudotuberculosis *FRC41, whereas the small contig was present in three tandem copies in the assembled chromosome (data not shown). The remaining gaps were closed by a PCR strategy that was supported by the related reference contig arrangement tool r2cat [13], using the *Corynebacterium diphtheriae *NCTC 13129 genome sequence as a reference [14]. The final assembly of the DNA sequences yielded a circular chromosome with a size of 2,337,913 bp and a mean G+C content of 52.2% (Figure 1A). Considering the final size of the *C. pseudotuberculosis *FRC41 chromosome, a 40-fold coverage was initially obtained by pyrosequencing.

The annotation of the *C. pseudotuberculosis *FRC41 genome sequence was performed with the GenDB software system [15] and resulted in the detection of 2,110 protein-coding regions. Furthermore, 49 tRNA genes were predicted by the tRNAscan-SE program [16] and four *rrn *operons were detected on the leading strands of the chromosome (Figure 1B). A plot of the calculated G/C skew [(G-C)/(G+C)] indicated a bi-directional replication mechanism of the *C. pseudotuberculosis *chromosome (Figure 1A). According to the presence and distribution of six conserved DnaA boxes, the *oriC *is located downstream of the *dnaA *coding region [17]. The G/C skew and the biased distribution of architecture imparting sequences (AIMS) on the leading and lagging strands indicated the presence of a *dif *region [18] at the expected position of 180° from *oriC*, dividing the chromosome of *C. pseudotuberculosis *FRC41 into two replichores of similar size (Figure 1B). Synteny analysis by reciprocal best matches with BLASTP [19] revealed a highly conserved order of orthologous genes between the chromosomes of *C. pseudotuberculosis *FRC41 and *C. diphtheriae *NCTC 13129 (Figure 1C), which is consistent with the close phylogenetic relationship of both species [1] and the observation that genetic rearrangements are rare in the genomes of species belonging to the main lineage of the genus *Corynebacterium *[20,21]. The calculated reciprocal best BLASTP hits [19] were used also to compare the predicted proteome of *C. pseudotuberculosis *FRC41 with the complete set of proteins encoded in the genome of *C. diphtheriae *NCTC 13129 [14]. This comparative content analysis at the proteome level revealed that 1610 proteins (76.3%) of *C. pseudotuberculosis *FRC41 share a homologue in the genome of *C. diphtheriae *NCTC 13129 (data not shown). The characteristic features of *C. pseudotuberculosis *FRC41 are apparently based on a distinct gene set, defining its lifestyle and pathogenicity, such as the *pld *gene encoding phospholipase D [22]. In the following sections, we describe a collection of relevant genes contributing to the lifestyle and pathogenicity of *C. pseudotuberculosis *FRC41 and deduce their integration into a transcriptional gene-regulatory network.

### The transcriptional regulatory repertoire of *C. pseudotuberculosis *FRC41

**Figure 2:**
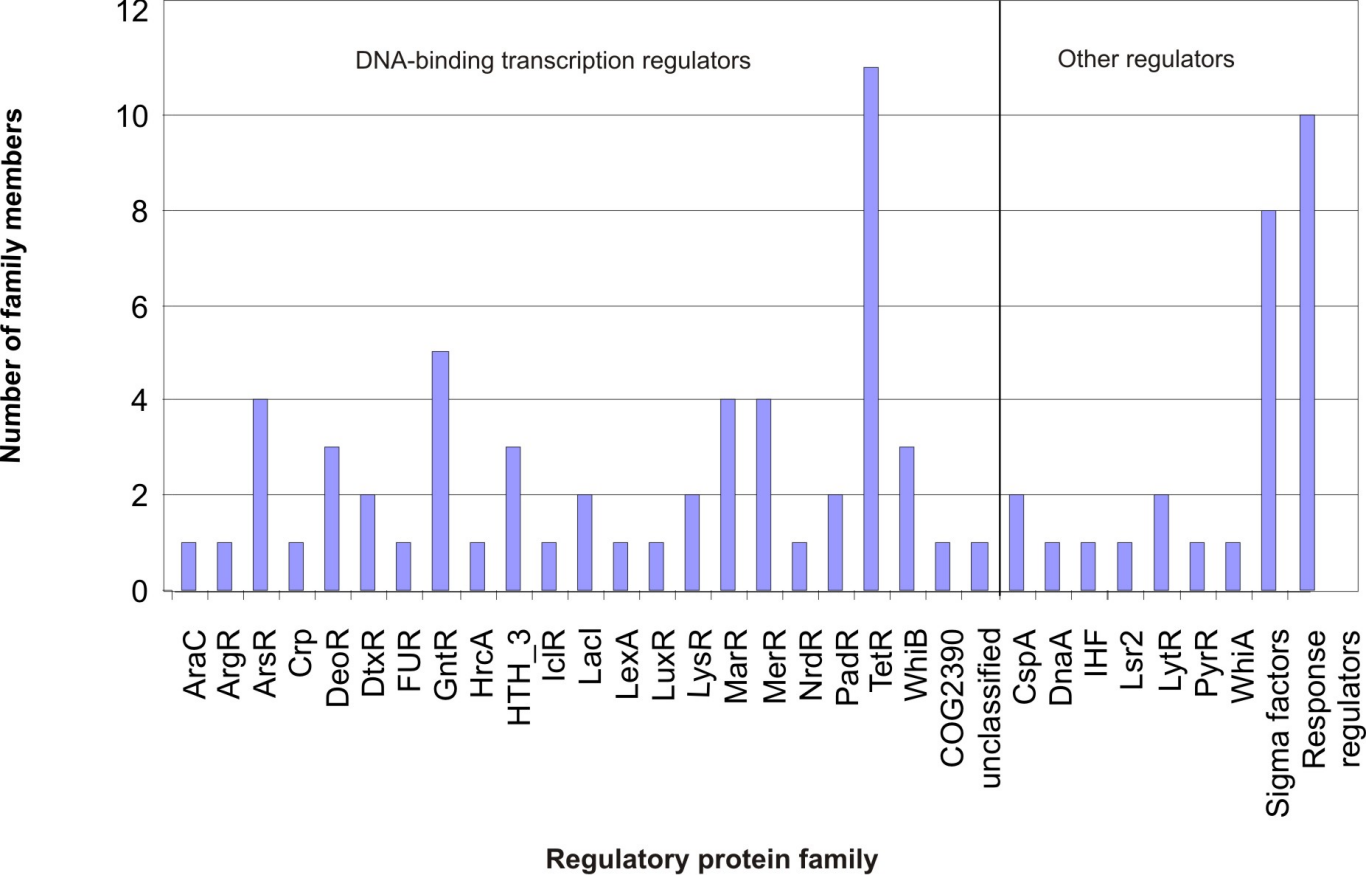
**Classification of the predicted transcriptional regulatory repertoire of *C. pseudotuberculosis *FRC41 into regulatory protein families**. The number of candidate transcription regulators assigned to a regulatory protein family is shown.

The repertoire of candidate transcription regulators encoded in the *C. pseudotuberculosis *FRC41 genome was deduced from the functional genome annotation taking into account the comprehensive knowledge of the reconstructed gene-regulatory network of *Corynebacterium glutamicum *ATCC 13032 [23]. A collection of 83 genes encoding DNA-binding transcription regulators, sigma factors and response regulators of two-component systems can be regarded as the minimal regulatory repertoire of *C. pseudotuberculosis *FRC41 (Figure 2). This set of candidate transcription regulators represents 3.9% of the predicted protein-coding genes of the *C. pseudotuberculosis *FRC41 genome. This value is in agreement with previous observations that less than 10% of the total number of predicted proteins are associated with transcriptional regulatory processes in bacteria [24] and is in the range known from other pathogenic corynebacteria, such as *C. diphtheriae *and *Corynebacterium jeikeium *[25]. The collection of potential transcription regulators was grouped into regulatory protein families according to their amino acid sequence similarities and domain organizations [26]. This bioinformatic classification assigned the candidate transcription regulators to 31 regulatory protein families, with one regulator (cpfrc_01413) remaining unclassified. The regulatory protein families detected in *C. pseudotuberculosis *FRC41 vary significantly in their number of representatives (Figure 2). The largest family of DNA-binding transcription regulators is TetR with 11 members, followed by GntR with 5 proteins. The TetR family of transcription regulators is widely distributed among bacterial species [27] and is also the most prevalent group of regulatory proteins in other corynebacteria [25]. It is noteworthy that the detected collection of transcription regulators includes only 23 out of the 24 proteins that hitherto constituted the core of DNA-binding transcription regulators in corynebacteria, as *C. pseudotuberculosis *FRC41 lacks a gene for an orthologue of the conserved TetR regulator MfsR (Cg0454) [23,25]. The knowledge of the transcriptional regulatory repertoire of *C. pseudotuberculosis *FRC41 was combined with regulons contributing to virulence by bioinformatic motif searches for DNA-binding sites of prominent regulatory proteins.

### Iron regulation in physiology and virulence of *C. pseudotuberculosis*

**Figure 3:**
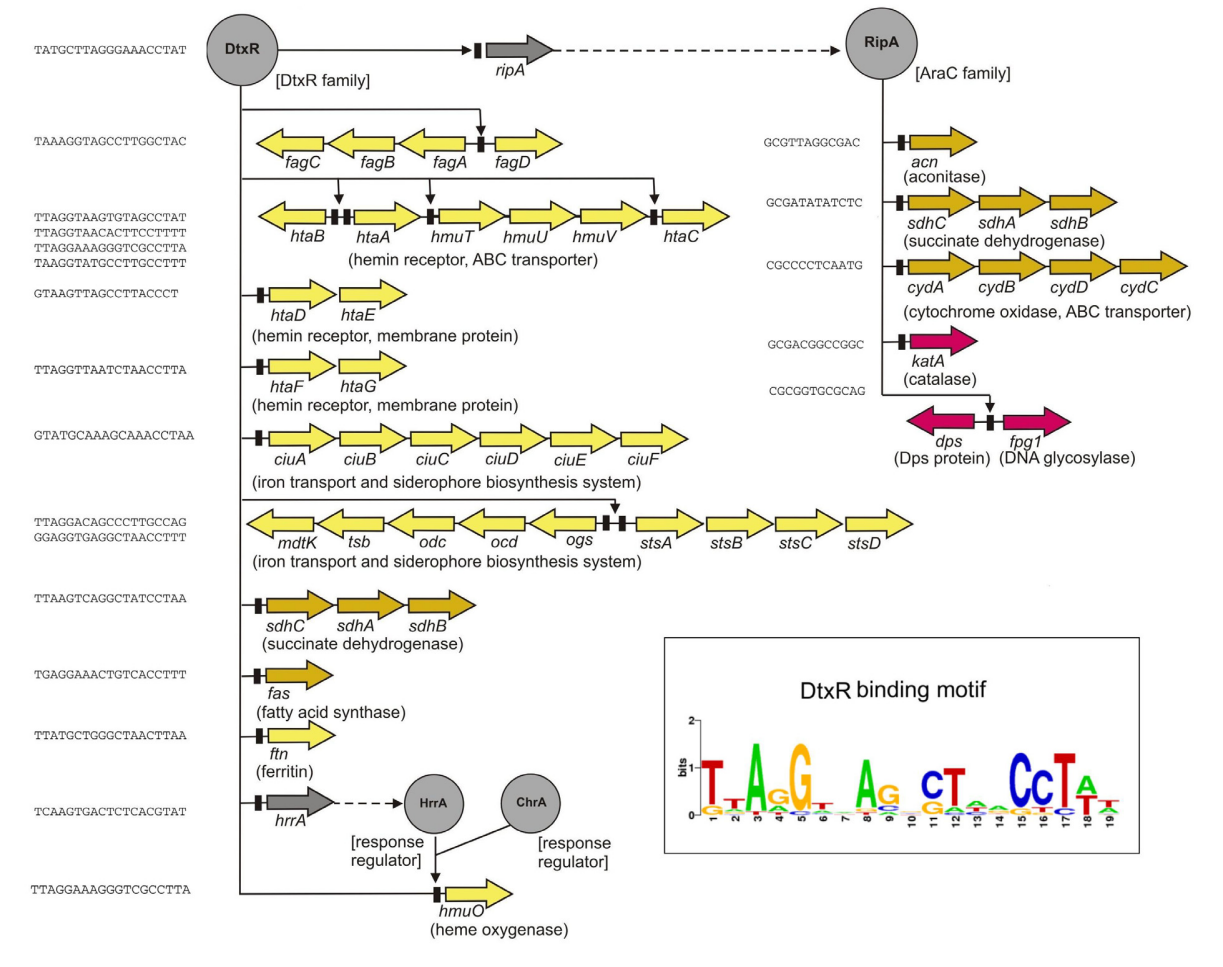
**Regulons involved in iron regulation of *C. pseudotuberculosis *FRC41**. The DtxR and RipA regulons controlling iron homeostasis and the respective gene-regulatory interactions were deduced from a genome-scale network transfer approach [114] and the combined use of hidden Markov models and position weight matrices [74]. The assignment of the transcription regulators into the regulatory protein families of *C. pseudotuberculosis *FRC41 is indicated. Predicted DNA-binding sites are listed by sequence and are shown as black boxes, regulated target genes are shown as arrows and coloured as follows: grey, regulatory gene; yellow, gene involved in iron uptake and iron storage; orange, gene involved in central metabolism; red, gene contributing to the oxidative stress response. The highly conserved coregulation of the *hmuO *gene by DtxR and the response regulators HrrA and ChrA in corynebacteria is indicated [23]. The 19-bp consensus sequence of the DtxR-binding site of *C. pseudotuberculosis *FRC41 is shown as DNA sequence logo. The 16 predicted DNA-binding sites of DtxR were used as input data for the WebLogo tool [115].

For most bacteria, iron is essential as a cofactor for proteins involved in important cellular functions, such as DNA biosynthesis and respiration [28]. Thus, iron acquisition is a vital function for bacterial survival. As iron limitation is a common strategy by which a mammalian host suppresses bacterial growth, iron has a decisive role in infectious diseases. On the one hand, pathogenic bacteria have to compete for iron in the host so that they can multiply and establish a successful infection. On the other hand, they must regulate iron metabolism to prevent excess iron that can initiate the generation of toxic oxygen radicals from normal products of metabolism by Fenton chemistry. Bacteria have solved the problem of iron acquisition and homeostasis by encoding a variety of high-affinity uptake systems that are tightly regulated at the transcriptional level [28]. The *fagCBA-fagD *genes of *C. pseudotuberculosis*, for instance, encode an iron uptake system that is regulated by iron *in vitro *[29]. A *fag *mutant showed a reduced virulence in a goat model of caseous lymphadenitis when compared with a wild-type control, although no defect in iron utilization by the mutant strain was determined [29]. As the expression of the *fagABCD *genes contributed to the virulence of *C. pseudotuberculosis *from animal sources, the complete genome sequence of FRC41 was screened for the presence of these genes, additional iron uptake systems and the responsible transcription regulator (Figure 3).

The *dtxR *gene of *C. pseudotuberculosis *FRC41 encodes a homologue of the diphtheria toxin repressor DtxR that is activated by iron and controls a complex gene-regulatory network involved in iron homeostasis in corynebacteria [30,31]. The detection of a putative DtxR-binding site in the *fagA*-*fagD *intergenic region supports the previously observed differential expression pattern of the *fagABC *operon *in vitro *[29]. We combined the functional annotation of the *C. pseudotuberculosis *FRC41 genome sequence with a bioinformatic motif search for DtxR-binding sites using a hidden Markov model and a position weight matrix with input data from the DtxR regulon of *C. glutamicum *[31]. In this way, additional genes were assigned to the DtxR regulon of *C. pseudotuberculosis *FRC41, including several gene clusters involved in the utilization of various host compounds as iron sources (Figure 3). One gene region revealed similarity to the hemin utilization system HmuTUV from *C. diphtheriae *[32]. The corresponding hemin binding protein HtaA is probably associated with the cell envelope and involved in the utilization of heme iron [33]. Two gene clusters assigned to the DtxR regulon of *C. pseudotuberculosis *FRC41 include *htaA*-like genes (*htaD *and *htaF*) that are associated with genes encoding membrane proteins (*htaE *and *htaG*), suggesting a role of these clusters in the acquisition of iron from the host (Figure 3). The *hmuO *gene of the DtxR regulon encodes heme oxygenase that releases iron from the protoporphyrin ring of heme and facilitates the acquisition of iron from heme and hemoglobin [34]. Because of the potential toxicity of both, iron and heme, the expression of *hmuO *in *C. diphtheriae *is under complex control, comprising the iron-responsive repressor DtxR and the heme-dependent activators ChrA and HrrA that are part of the two-component signal transduction systems ChrA-ChrS and HrrA-HrrS [35,36]. A BLAST search across the *C. pseudotuberculosis *FRC41 genome revealed two response regulators that share similarity with ChrA and HrrA. As the *hrrA *gene of *C. pseudotuberculosis *FRC41 is part of the DtxR regulon, a complex hierarchical control of *hmuO *gene expression might be established in this bacterium (Figure 3).

Iron acquisition can moreover involve the synthesis and secretion of high-affinity iron chelators, termed siderophores, which are synthesized by nonribosomal peptide synthetases or by biosynthesis pathways independent of these multimodular enzymes [37,38]. The genome of *C. pseudotuberculosis *FRC41 contains two DtxR-regulated gene clusters that are probably associated with pathways for siderophore biosynthesis independent of nonribosomal peptide synthetases (Figure 3). The *ciu *locus comprises the *ciuABCD *(ABC-type transporter), *ciuE *(siderophore biosynthesis-related protein) and *ciuF *(putative efflux protein) genes. The predicted product of the *ciuE *gene is similar to aerobactin biosynthesis enzymes [39]. The expression of the *ciuA *gene encoding the lipoprotein receptor of the ABC transport system has been detected *in vivo *by a reporter transposon system [40]. The *ciu *gene region detected in the genome of *C. pseudotuberculosis *FRC41 is similar to the *ciu *gene locus from *C. diphtheriae *NCTC 13129, with the exception that it lacks the *ciuG *gene, encoding a protein of unknown function [41].

The second DtxR-regulated gene cluster related to siderophore biosynthesis and excretion includes four genes probably constituting the biosynthesis pathway (*ogs*, *ocd*, *odc *and *tsb*), a gene encoding an efflux protein (*mdtK*) and four genes encoding an ABC-type transporter (*stsABCD*) (Figure 3). Ornithine cyclodeaminase (*ocd*) and ornithine decarboxylase (*odc*) as well as monooxygenase (*ogs*) and synthetase (*tsb*) functions encoded in this gene cluster are components of widely distributed routes for siderophore biosynthesis [39]. Additional DtxR-binding sites were detected in front of *ftn *(ferritin) and *fas *(fatty acid synthase) and the *sdhCAB *(succinate dehydrogenase) operon that are also part of the DtxR regulon in *C. glutamicum *[31,42]. Ferritins act primarily in iron storage and are thus central to the natural regulation of iron in the bacterial cell [28].

Furthermore, the *ripA *gene encoding a DNA-binding transcription regulator of the AraC protein family was assigned to the DtxR regulon of *C. pseudotuberculosis *FRC41 (Figure 3). The expression of the orthologous *ripA *gene from *C. glutamicum *is also controlled directly by DtxR [42]. Under iron limitation, the RipA protein acts as a repressor of genes coding for iron proteins in *C. glutamicum *[42]. Candidate RipA-binding sites were detected by bioinformatic pattern searches in the genome sequence of *C. pseudotuberculosis *FRC41 in front of the *acn *(aconitase) gene and upstream of the *sdhCAB *(succinate dehydrogenase) and *cydABDC *operons (cytochrome *bd*-type menaquinol oxidase and ABC-type transporter), thereby linking the availability of iron with the expression of genes in the citrate cycle and the respiratory energy metabolism of *C. pseudotuberculosis*. Due to its high oxygen affinity, the cytochrome *bd *oxidase is used in many bacteria under microaerobic growth conditions [43]. Additional RipA-binding sites were detected in front of the *katA *(catalase) gene and in the *dps*-*fpg1 *(DNA protection during starvation protein, formamidopyrimidine-DNA glycosylase) intergenic region, integrating protective functions into the RipA regulon (Figure 3). Dps-like proteins effectively protect DNA against oxidizing agents by nonspecific DNA-binding and physical sequestration that limits DNA accessibility to detrimental factors [44]. They also act as iron-binding and storage proteins and catalyze the oxidation of ferrous iron to ferric iron by H_2_O_2_, which prevents the formation of hydroxyl radicals by the Fenton reaction [45]. The *lsr2 *gene of *C. pseudotuberculosis *FRC41 encodes the multifunctional histone-like protein and transcription regulator Lsr2 (Figure 2) that shares a number of physical properties with Dps-like proteins and the ability to bind DNA sequences with little specificity [46]. Like Dps, the Lsr2 protein may physically protect corynebacterial DNA against reactive oxygen intermediates [47]. Formamidopyrimidine-DNA glycosylase is a primary participant in the repair of DNA lesions caused by oxidative damage [48]. A second gene (*fpg2*) encoding formamidopyrimidine-DNA glycosylase was detected in the genome of *C. pseudotuberculosis *FRC41, but the upstream region of *fpg2 *apparently lacks a RipA-binding site. Catalase is another important protective component in the bacterial oxidative stress response which is involved in the detoxification of H_2_O_2 _[49]. Hence, the gene composition of the DtxR-RipA gene-regulatory network revealed the interdependence of iron metabolism and oxidative stress response and the regulatory connection between distinct physiological functionalities of the corynebacterial cell, including an important role in virulence of *C. pseudotuberculosis*.

### Manganese and zinc regulation in physiology and virulence of *C. pseudotuberculosis*

**Figure 4:**
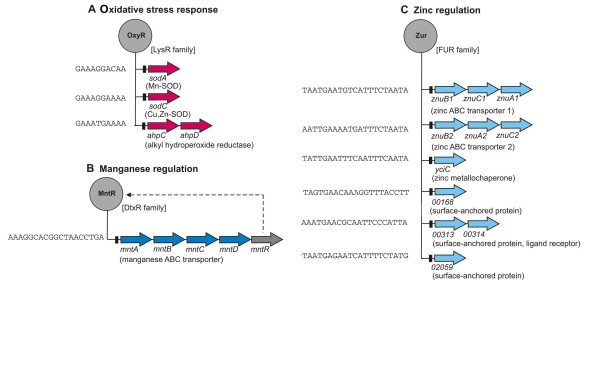
**Regulons involved in the oxidative stress response and in zinc and manganese regulation of *C. pseudotuberculosis *FRC41**. **(A)**, The OxyR regulon controlling the oxidative stress response. **(B)**, The MntR operon controlling the uptake of manganese. **(C)**, The Zur regulon controlling zinc metabolism. The assignment of the transcription regulators into the regulatory protein families of *C. pseudotuberculosis *FRC41 is indicated. The regulatory interactions were deduced from a genome-scale network transfer approach [114] and the combined use of a hidden Markov model and a position weight matrix [74]. Predicted DNA-binding sites are shown as black boxes and indicated by DNA sequence. The regulated target genes are shown as arrows and coloured as follows: grey, regulatory gene; red, gene contributing to the oxidative stress response; dark blue, gene involved in maganese uptake; light blue, gene involved in zinc metabolism.

Peroxynitrite and other reactive nitrogen and oxygen intermediates are produced by macrophages as part of their antimicrobial response [50]. Consequently, many pathogenic bacteria have evolved protection mechanisms against these reactive nitrogen and oxygen intermediates that have potent antimicrobial activity [51]. Four genes encoding protective enzymes probably involved in corresponding detoxification reactions were identified in the genome sequence of *C. pseudotuberculosis *FRC41, including alkyl hydroperoxide reductase (*ahpCD*), manganese-dependent superoxide dismutase (*sodA*) and copper, zinc-dependent superoxide dismutase (*sodC*). A bioinformatic pattern search with actinobacterial OxyR-binding sites as input data revealed that these genes are most likely under transcriptional control by OxyR (Figure 4A). The *oxyR *gene of *C. pseudotuberculosis *FRC41 is linked to the *ahpCD *genes and its gene product may act as a repressor of gene expression [52]. AhpC is a member of a large family of peroxidases that contribute to the antioxidant defense in bacteria [53]. The AhpC protein directly reduces peroxides and is in turn reduced by AhpD [54]. The mycobacterial AhpC protein also catalyzes the rapid conversion of peroxynitrite to nitrite to avoid the formation of deleterious nitrogen dioxide and hydroxyl radicals [55]. Superoxide dismutase converts superoxide anions into molecular oxygen and H_2_O_2_, the latter being broken in turn to H_2_O by the enzymatic activity of catalase [56]. Superoxide dismutases were classified into three evolutionarily distinct families according to the type of metal cofactors. Most bacteria possess either a manganese-dependent (Mn-SOD) or an iron-dependent (Fe-SOD) superoxide dismutase in their cytoplasm, while secreted copper, zinc-dependent superoxide dismutases (Cu,Zn-SODs) have been detected in pathogenic or endosymbiontic bacteria [57]. The genome of *C. pseudotuberculosis *FRC41 encodes two types of superoxide dismutases, a cytoplasmic Mn-SOD (SodA) and a secreted Cu,Zn-SOD (SodC) that is characterized by a lipobox motif and may be anchored in the cell membrane [58]. The extracellular location of SodC suggests that it may protect the surface of *C. pseudotuberculosis *cells against superoxide generated externally by the mammalian host cells. Likewise, the mycobacterial SodC protein contributes to the resistance of *Mycobacterium tuberculosis *against oxidative burst products generated by activated macrophages [59,60]. The protective activity of Cu,Zn-SODs has been associated with virulence in many bacteria, such as *Neisseria meningitidis *and *Haemophilus ducreyi *[61,62]. However, further experimental work is necessary to elucidate which protective enzyme contributes to the virulence of *C. pseudotuberculosis*.

As manganese and zinc ions are apparently involved as cofactors in the oxidative stress response of pathogens, the corresponding regulons involved in metal ion uptake were identified in the genome sequence of *C. pseudotuberculosis *FRC41 (Figure 4B). The uptake of manganese in *C. pseudotuberculosis *FRC41 is mediated by a typical manganese ABC-type transport system (*mntABCD*) that is negatively controlled at the transcriptional level by the metalloregulator MntR (Figure 4B). The manganese ABC transporter is composed of: a lipoprotein receptor (MntA), anchored to the cell membrane and functioning as an extracellular cation-binding protein; a cytoplasmic ATP-binding protein (MntB); and two integral membrane proteins (MntC and MntD) that mediate the cation flux [63]. A homologous gene cluster and its manganese-dependent transcriptional control by the metalloregulator MntR have been examined in the genome of *C. diphtheriae *[64]. The uptake of zinc ions in *C. pseudotuberculosis *FRC41 is probably mediated by two ABC transport systems (*znuB1C1A1 *and *znuB2A2C2*), as both gene regions are specified by the presence of Zur-binding sites (Figure 4C). The Zur protein is a metalloregulator of the ferric uptake regulator (FUR) family of DNA-binding transcription regulators [65,66]. The *znu *ABC transporter genes are key components of actinobacterial Zur regulons and their expression is generally repressed by Zur in a zinc-dependent manner [66]. Zinc resistance might be facilitated in *C. pseudotuberculosis *FRC41 by the ArsR-type transcription regulator Znr that probably controls the expression of the *czcE *gene encoding a cobalt/zinc/cadmium efflux system [23,67,68]. The *yciC *gene coding for a putative P-loop GTPase of the COG0523 protein family is also part of the Zur regulon in *C. pseudotuberculosis *FRC41 (Figure 4C). The YciC protein may function as a metallochaperone/insertase to enable the *in vivo *assembly of zinc-containing proteins under environmental conditions of zinc deficiency [69].

Moreover, the genome-wide motif search for Zur-binding sites in *C. pseudotuberculosis *FRC41 detected three genes (cpfrc_00168, cpfrc_00313, cpfrc_02059) encoding proteins with a carboxy-terminal sorting (LPxTG) signal that is generally used by Gram-positive bacteria to anchor proteins to the cell wall [70]. The Cpfrc_00168 protein contains two CnaB-like domains that may be involved in the positioning of a ligand binding domain away from the corynebacterial cell surface and is encoded adjacent to a putative sialoprotein-binding protein. The secreted proteins Cpfrc_00313 and Cpfrc_02059 contain actinobacterial surface-anchored protein domains for their covalent attachment to the cell wall [56]. The Cpfrc_00313 protein is encoded next to the components of a transporter and may act together with the Cpfrc_00314 protein as a substrate receptor for this system. Hence, the reconstruction of regulons participating in metal ion uptake of *C. pseudotuberculosis *FRC41 led to the detection of genes that may fulfill novel functions in sensing the presence of zinc in the environment.

### Genes encoding adhesive pili in *C. pseudotuberculosis *FRC41

**Figure 5:**
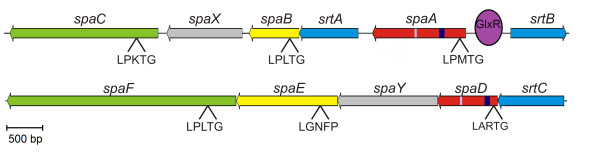
**Gene regions encoding adhesive pili of *C. pseudotuberculosis *FRC41**. The gene clusters involved in the synthesis of adhesive (Spa-like) pili of *C. pseudotuberculosis *FRC41 are shown. The gene clusters encode sortases required for the assembly of the pilus (blue), major pilins (red), minor pilins (yellow), pilus tip proteins (green), and proteins of unknown function (grey). The detected sorting (LPxTG) signals are indicated. Specifically marked in the major pilin proteins are the characteristic pilin boxes (blue) and E-boxes (white). The predicted binding of the transcription regulator GlxR in the *spaA*-*srtB *intergenic region is shown.

The complete set of predicted protein-coding regions of *C. pseudotuberculosis *FCR41 was subsequently screened for the presence of further proteins containing a typical sorting signal. This approach revealed ten additional proteins without any conserved domain organization [56] that were annotated as hypothetical proteins with LPxTG motif and, more interestingly, six proteins showing similarity to subunits of adhesive pili from *C. diphtheriae *NCTC 13129 (Figure 5). The corresponding coding regions are organized in two gene clusters that include sortase genes involved in the process of pilus polymerization [71,72]. The housekeeping sortase gene (cpfrc_02014; *srtD*) of *C. pseudotuberculosis *FCR41, necessary for the cell wall anchoring of pilin monomers and pilus polymers [73], is located elsewhere in the chromosome. The adhesive pili of *C. pseudotuberculosis *FCR41 consist of major pilin subunits (SpaA, SpaD), minor pilin subunits (SpaB, SpaE) and tip proteins (SpaC, SpaF) that are characterized by conserved amino acid motifs (Figure 5). The function of the hypothetical proteins SpaX and SpaY encoded in the *spa *gene regions of *C. pseudotuberculosis *FCR41 is currently unknown. According to alkaline phosphatase (*phoZ*) gene fusions generated with a reporter transposon system in *C. pseudotuberculosis*, at least the SpaABC pilus is expressed in this species [1]. The *spaABC *gene cluster contains a putative DNA-binding site for the cAMP-sensing transcription regulator GlxR in the *spaA*-*srtB *intergenic region (Figure 5), thereby connecting the expression of pilus genes with a global gene-regulatory network in *C. pseudotuberculosis *[74,75]. The adhesive pili of *C. diphtheriae *NCTC 13129 are covalently anchored to the cell wall and can mediate the initial adhesion to host tissues and other bacterial cells [76]. The adherence of *C. diphtheriae *to pharyngeal epithelial cells is mediated also by the minor pilin of the adhesive pilus, as this pilin subunit is covalently anchored to the cell wall and can provide tight contact between the bacterial cell and the host tissue in the absence of a pilus shaft [77]. Considering a similar functioning of the predicted SpaABC and SpaDEF pilus proteins from *C. pseudotuberculosis*, either a complete pilus structure or the minor pilins SpaB and SpaE can probably make the initial contact with host cell receptors to enable additional ligand-receptor interactions and to facilitate the efficient delivery of virulence factors and intracellular invasion.

### Candidate virulence factors of *C. pseudotuberculosis *FRC41 and their integration into a gene-regulatory network

The observation that adhesive pili promote the adherence of *C. diphtheriae *to host tissue suggests that the SpaABC and SpaDEF pili from *C. pseudotuberculosis *FRC41 can be regarded as potential virulence factors [77]. To extend the view on proteins contributing to the pathogenicity of *C. pseudotuberculosis *FRC41, the genome sequence was screened for further candidate genes encoding virulence factors (Table 1). The major virulence factor of *C. pseudotuberculosis *is the sphingomyelin-degrading phospholipase D that facilitates the persistence and spread of the bacterium within the host [5,6]. The expression of the *pld *gene is regulated by multiple environmental stimuli, including heat, and plays a role in the reduction of macrophage viability following infection [78,79]. The secreted corynebacterial protease CP40 was detected previously as a protective antigen of *C. pseudotuberculosis *and shown to be of the serine protease type, although BLAST searches revealed homology to endoglycosidases [80]. The extracellular CP40 enzyme may contribute to the virulence of *C. pseudotuberculosis *by its proteolytic activity, but the enzymatic activity of CP40 was not detectable in culture supernatants [80]. However, vaccination of sheep with this antigen resulted in protection against infection with *C. pseudotuberculosis*, probably by affecting directly the function of the CP40 protein and indirectly the growth of the pathogen [11]. The genome sequence of *C. pseudotuberculosis *FRC41 revealed three additional genes encoding secreted proteases, including two subtilisin-like serine proteases and one trypsin-like serine protease (Table 1). Extracellular proteases may exhibit a wide range of pathogenic potentials when interacting with the defense mechanisms and tissue components of the host. Redundant enzymatic systems are moreover suitable to promote the survival of pathogens under unfavorable environmental conditions encountered in the infected host [81]. The genome sequence of *C. pseudotuberculosis *FRC41 also encodes a secreted protein of the SGNH-hydrolase subfamily (Table 1). SGNH-hydrolases are a diverse family of lipases and esterases which are known to act as virulence factors in other bacteria, such as *Streptomyces scabies*, the causal agent of the potato scab disease [56]. Further experimental studies are required to elucidate whether the expression of the secreted enzymes promotes the virulence of *C. pseudotuberculosis *FRC41.

**Table 1:** Candidate determinants contributing to virulence of *C. pseudotuberculosis* FRC41

Identifier	Gene	Predicted protein function
cpfrc_00029	*pld*	phospholipase D (sphingomyelin-degrading enzyme)
cpfrc_01895	*cpp*	corynebacterial protease CP40 (serine protease)
cpfrc_00397	-	secreted subtilisin-like serine protease
cpfrc_01634	-	secreted subtilisin-like serine protease
cpfrc_00562	-	secreted trypsin-like serine protease
cpfrc_00536	-	secreted SGNH-hydrolase
cpfrc_00386	*nanH*	neuraminidase H (sialidase)
cpfrc_01079	*rpfI*	resuscitation-promoting factor interacting protein (D,L-endopeptidase)
cpfrc_00594	*rpfA*	resuscitation-promoting factor A (muralytic enzyme)
cpfrc_00679	*rpfB*	resuscitation-promoting factor B (muralytic enzyme)
cpfrc_00128	*nor*	nitric oxide reductase
cpfrc_00565	*nrpS1*	nonribosomal peptide synthetase 1
cpfrc_01801	*nrpS2*	nonribosomal peptide synthetase 2
cpfrc_00492	*dtsR1*	acetyl-CoA carboxylase β-subunit involved in fatty acid synthesis
cpfrc_00491	*dtsR2*	acyl-CoA carboxylase β-subunit involved in mycolic acid synthesis
cpfrc_01953	*accD3*	acyl-CoA carboxylase β-subunit involved in mycolic acid synthesis

**Figure 6:**
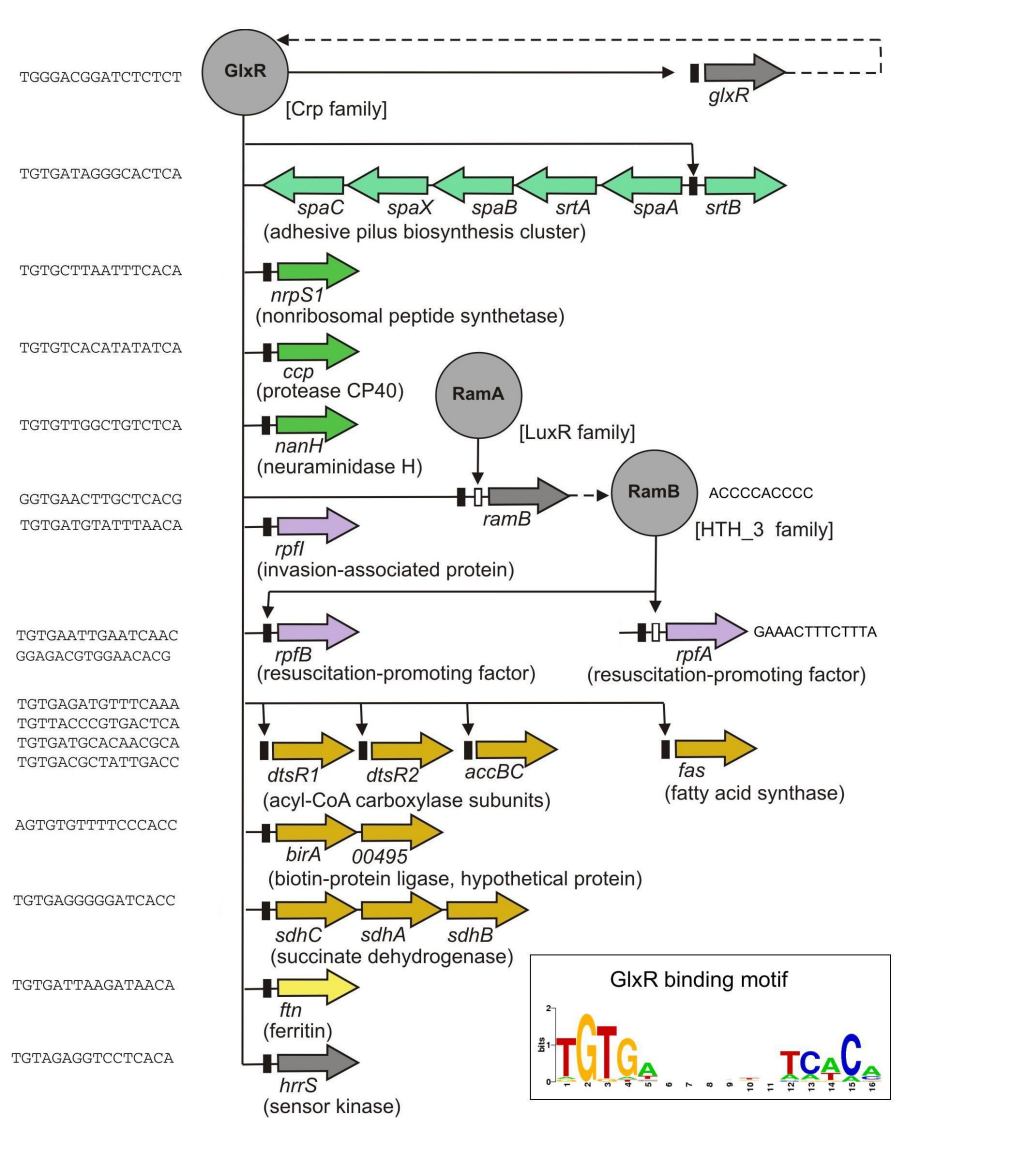
**Regulatory interactions involved in the control of potential virulence factors of *C. pseudotuberculosis *FRC41**. Transcription regulators controlling the expression of candidate virulence factors are shown. The regulatory interactions were deduced from a genome-scale network transfer approach [114] and the combined use of a hidden Markov model and a position weight matrix [74]. The assignment of the transcription regulators into the regulatory protein families of *C. pseudotuberculosis *FRC41 is indicated. Predicted DNA-binding sites of RamA and RamB are shown as white boxes and predicted DNA-binding sites of GlxR as black boxes. Regulated target genes are shown as arrows and coloured as follows: grey, regulatory gene; yellow, gene involved in iron storage; orange, gene of central metabolism or fatty acid/mycolic acid biosynthesis; violet, gene involved in the resuscitation process; light green, gene involved in the assembly of adhesive pilus; dark green, other potential virulence factor gene. The 16-bp consensus sequence of the GlxR-binding site of *C. pseudotuberculosis *FRC41 is shown as DNA sequence logo. The 17 predicted DNA-binding sites of GlxR were used as input data for the WebLogo tool [115].

Another candidate virulence factor of *C. pseudotuberculosis *FRC41 is the extracellular neuraminidase NanH (Table 1). Neuraminidases, or sialidases, belong to a class of glycosyl hydrolases that catalyze the removal of terminal sialic acid residues from a variety of glycoconjugates and can contribute to the recognition of sialic acids exposed on host cell surfaces [82,83]. The homologous counterpart of NanH was recently characterized in *C. diphtheriae *KCTC3075 and shown to be a protein containing neuraminidase and *trans*-sialidase activities [84]. *Trans*-sialidases located on the bacterial cell surface can be used for the decoration of sugar moiety acceptors with sialic acid to enable the invasion of hosts under certain conditions. The *trans*-sialidase activity is of importance for many pathogenic bacteria and the corresponding proteins are therefore considered potential virulence factors [82]. Iron limitation reduced the number of sialic acid residues on the surface of *C. diphtheriae *cells and their adhesive properties, indicating that the expression of dissimilar virulence determinants is coordinately controlled by a gene-regulatory system [85]. By bioinformatic pattern searches, a GlxR-binding site was detected in the upstream region of the *nanH *gene of *C. pseudotuberculosis *FRC41, suggesting that the cAMP-sensing transcription regulator GlxR might be involved in the control of this virulence factor gene (Figure 6).

Likewise, a DNA-binding site for GlxR was detected in front of the *rpfI *gene encoding an invasion-associated protein that is involved in cell surface organization and adhesion of corynebacteria [86]. The homologue of RpfI in *M. tuberculosis *(named RipA) revealed endopeptidase activity and interacts with the resuscitation-promoting factor RpfB, representing a peptidoglycan glycosidase [86,87]. Two genes (*rpfA *and *rpfB*) encoding resuscitation-promoting factors are present in the genome of *C. pseudotuberculosis *FRC41 (Table 1). Important roles in pathogenesis for peptidoglycan hydrolytic enzymes have been proposed [88] and an analogous system combining the activities of a muramidase and an endopeptidase contributed to the virulence of *Listeria monocytogenes *[89]. As previously demonstrated in *C. glutamicum *[74,90], the expression of *rpfI*, *rpfA *and *rpfB *in *C. pseudotuberculosis *FRC41 is probably under complex control by three regulatory proteins, GlxR, RamB and RamA (Figure 6).

Another potential virulence factor of *C. pseudotuberculosis *FRC41 is represented by the *nor *gene encoding nitric oxide reductase (Table 1). This enzyme is generally involved in the detoxification of nitric oxide and consequently necessary for the long-term persistence of pathogens in macrophages [91]. The toxic properties of nitric oxide are used by the host immune system to kill or slow down the growth of pathogenic bacteria [51]. Interestingly, the expression of the *nor *gene was not induced upon the infection of macrophages by animal *C. pseudotuberculosis *[4]. As the expression of *nor *is typically activated by a transcription regulator in response to the presence of nitric oxide [92], the regulatory pattern of *nor *transcription and its contribution to the protection against nitric oxide remains unclear.

The previous search for macrophage-induced genes of animal *C. pseudotuberculosis *by means of a cloned promoter library provided two gene tags showing significant induction rates in macrophages [4]. The nucleotide sequence of the respective gene tags revealed similarity to nonribosomal peptide synthetases (44-fold induction) and to the β-subunit of acyl-CoA carboxylases (24-fold induction), respectively. The genome sequence of *C. pseudotuberculosis *FRC41 encodes two nonribosomal peptide synthetases, NrpS1 and NrpS2 (Table 1). These modular enzymes are used by microorganisms to participate in the synthesis of many secondary metabolites, including for instance siderophores and antibiotics [93]. As both *nrpS *genes were not assigned to the iron-responsive DtxR regulon of *C. pseudotuberculosis *FRC41 and siderophore biosynthesis is carried out by alternative pathways independent of nonribosomal peptide synthetases, a physiological role in iron metabolism of the two proteins cannot be deduced from the current data. However, the strong upregulation of gene expression in macrophages points toward a protective or toxic function during the infection of at least one nonribosomal peptide synthetase [4]. A role in virulence of a secondary metabolite produced by a nonribosomal peptide synthetase has been demonstrated in *Streptomyces acidiscabies*. This phytopathogen produces thaxtomin A which is necessary for the infection of potato tubers [94].

Three genes coding for β-subunits of acyl-CoA carboxylases are present in the genome of *C. pseudotuberculosis *FRC41 (Table 1). These genes are located in highly conserved regions of corynebacterial genomes and are essential for either fatty acid synthesis (*dtsR1*) or mycolic acid synthesis (*dtsR2 *and *accD3*) [95]. The acetyl-CoA carboxylase of *C. glutamicum *consists of the biotinylated α-subunit AccBC, the β-subunit AccD1 (DtsR1) and the small AccE protein. The acyl-CoA carboxylase involved in mycolic acid synthesis of *C. glutamicum *consists of the two β-subunits AccD2 and AccD3 (DtsR2 and AccD3) in addition to AccBC and AccE [95,96]. The expression of the respective genes in *C. pseudotuberculosis *FRC41 is probably controlled by the global transcription regulator GlxR (Figure 6), providing hints that this regulatory protein has a key function in connecting both the central metabolism and the expression of candidate virulence factors in this pathogenic bacterium. Mycolic acids, which are long-chain α-alkyl-β-hydroxy fatty acids, are important components of the corynebacterial cell wall [97] and probably associated with the pathogenicity of *C. pseudotuberculosis *[98]. They can provide a thick layer at the outer surface of the cell that protects the bacterium from antibiotics and the host's immune system [99]. Moreover, the noncovalently bound trehalose dimycolate is a well-established immunostimulatory compound with toxic properties [97]. Variations in the amount of mycolic acids and differences in other cell surface properties provide a basis for explaining the aggregation capacity of *C. pseudotuberculosis *in suspension, the strong aggregation within macrophages, the formation of thick capsules after the release of the pathogen from macrophages [11], and the occurrence of virulent and attenuated strains [98].

## Conclusions

The complete genome sequence of *C. pseudotuberculosis *FRC41 of human origin provides detailed insights into the gene repertoire contributing to the virulence of this bacterium that was isolated from a rare case of necrotizing lymphadenitis [12]. By combining *in silico *data obtained from the genome annotation with previous experimental knowledge, occasional observations on genes that affect the virulence of *C. pseudotuberculosis *were integrated into a global view on the pathogenicity of this species. The reconstruction of the DtxR regulon for instance provides a comprehensive set of genes involved in the acquisition of iron and extends the initial observation that a *fagB *mutant was not impaired in iron uptake [29]. A systematic mutational characterization of the newly detected iron transporters and siderophore biosynthesis gene clusters may help to dissect the contribution of each system to the virulence of *C. pseudotuberculosis *FRC41. It is very likely that the *ciu *siderophore biosynthesis and transport system may complement the uptake of iron by the FagABCD transporter under certain environmental conditions, as the *ciuA *gene was shown earlier to be expressed in *C. pseudotuberculosis *[1]. Likewise, a reporter transposon system indicated that a gene coding for a fimbrial subunit is expressed during growth of *C. pseudotuberculosis *in standard medium [40]. As the genome sequence of *C. pseudotuberculosis *FRC41 revealed two gene clusters encoding adhesive pili, it is interesting to examine how the respective pilin monomers or pilus polymers contribute to the adherence of *C. pseudotuberculosis *to host tissue and how their expression is controlled. We detected a DNA-binding site for the cAMP-sensing transcription regulator GlxR in the *spaA*-*srtB *intergenic region, whereas the iron-responsive regulator DtxR was proposed to control the assembly of pilin subunits in *C. diphtheriae *NCTC 13129 [100]. Another previous study provided two DNA sequence tags for macrophage-induced genes [4] that were linked to the nonribosomal peptide synthetase genes *nrpS1 *and *nrpS2 *and to the acyl-CoA carboxylase subunit genes *dtsR1*, *dtsR2 *and *accD3 *by means of the genome sequence of *C. pseudotuberculosis *FRC41. The detection of the latter genes is consistent with the observation that the cell surface of *C. pseudotuberculosis *is an important factor contributing to virulence [98]. Future work should compare the knowledge deduced from the genome sequence of *C. pseudotuberculosis *FRC41 with the genetic information generated from other human isolates or animal pathogens of the different biovars [101], or even extend the genome comparison to the pangenomic level. This global comparative approach with a larger set of sequenced genomes may provide comprehensive insights into the distinctive features of each biovar or strain. As the currently available commercial vaccines are unable to fully protect susceptible animals [102,103], the extended knowledge of potential virulence factors and novel antigens of *C. pseudotuberculosis *might be helpful for the design of more effective vaccines and molecular diagnostics to control caseous lymphadenitis in sheep and goats to reduce thereby the occupational risk exposure for humans.

## Methods

### Bacterial strain and growth conditions

*C. pseudotuberculosis *FRC41 was isolated from the inguinal lymph node of a 12-year-old French girl with necrotizing lymphadenitis [12]. The taxonomic identification of this clinical isolate was recently confirmed using multiplex PCR and the nucleotide sequence of the *pld *gene (N. Guiso, unpublished data). *C. pseudotuberculosis *FRC41 was routinely grown in brain-heart-infusion (BHI) broth or on Columbia agar with sheep blood at 37°C.

### Preparation of chromosomal DNA for genome sequencing

The preparation of chromosomal DNA from *C. pseudotuberculosis *FRC41 was performed as follows: 50-ml aliquots of cultures grown for 48-72 h were centrifuged at 4°C and 2,000 × *g *for 20 min. The cell pellets were resuspended in 0.6 ml Tris/NaCl buffer [10 mM Tris (pH 7.0), 10 mM EDTA (pH 8.0), 300 mM NaCl] and transferred to VK01 Precellys lysing tubes. The cells were lysed by means of a Precellys 24-Dual Tissue Homogenizer, using two cycles of 6,500 rpm for 15 sec with an interval of 30 sec. The chromosomal DNA was purified by phenol/chloroform/isoamyl alcohol (25:24:1) extraction and precipitated with ethanol. The DNA concentration was determined with a Tecan Infinite 200 Microplate Reader.

### Sequencing of the *C. pseudotuberculosis *FRC41 genome

A total of 5 μg of purified chromosomal DNA was used for constructing a single-stranded template DNA library. The DNA concentration of the library was measured by using the Agilent RNA 6000 Nano Kit. The preparation of the single-stranded template DNA library and DNA sequencing were performed according to manufacturer's protocols (Roche Applied Science). The sequencing of *C. pseudotuberculosis *DNA was carried out with the Genome Sequencer FLX Instrument and Titanium chemistry (Roche Applied Science). The sequence data were assembled with the GS de novo Assembler Software (Version 2.3). According to the 454 Newbler Metrics file, 286,938 reads representing 94,447,635 bases were assembled. Using the default cutoff of 500 bases for the size classification of the contigs, ten large contigs (≥ 500 bases) and one small contig (313 bases) were obtained to give a total size of 2,319,243 bases. The gap closure process was supported by the related reference contig arrangement tool r2cat, using the *C. diphtheriae *NCTC 13129 genome sequence as a reference [13]. The remaining gaps in the genome sequence were closed by PCR with Phusion hot start high-fidelity DNA polymerase (Finnzymes) and genomic template DNA. All primers used in this study were synthesized by Metabion. The PCR assays were carried out with a TProfessional PCR thermocycler (Biometra) according to standard protocols (Finnzymes). The amplified DNA fragments linking the individual contigs were sequenced by IIT Biotech. All DNA sequences were uploaded into the Consed program [104] to generate the complete genome sequence of *C. pseudotuberculosis *FRC41.

### Bioinformatic analysis of the complete genome sequence

The assembled sequence of *C. pseudotuberculosis *FRC41 was uploaded into the bacterial genome annotation system GenDB [15]. The annotation of the complete genome sequence was performed as described previously [105], followed by manual curation. Analyses of the predicted gene content and the metabolic properties of *C. pseudotuberculosis *FRC41 were accomplished by the computer programs EDGAR [19] and CARMEN [106]. The synteny between the genomes of *C. pseudotuberculosis *FRC41 and *C. diphtheriae *NCTC 13129 was calculated by the EDGAR software [19]. The origin of chromosomal DNA replication was predicted with the Ori-Finder tool [17]. The genome sequence of *C. pseudotuberculosis *FRC41 has been deposited in the GenBank database with accession number CP002097.

### Bioinformatic analysis of the regulatory repertoire

The detection of the transcriptional regulatory repertoire of *C. pseudotuberculosis *FRC41 was performed by a combined bioinformatic approach using several tools and programs [26]. Proteins containing putative DNA-binding domains were detected by means of the HMM library and genome assignments server Superfamily version 1.75 [107]. To identify among the set of potential DNA-binding proteins those representing transcription regulators, hidden Markov model (HMM) profiles of regulatory protein families were downloaded from the Pfam database version 24.0 [108] and used for searches against the predicted *C. pseudotuberculosis *FRC41 proteins by applying the HMMsearch module of the profile hidden Markov model software HMMER [109]. Moreover, the helix-turn-helix (HTH) recognition tool [110] integrated in the GenDB platform was applied to scan the putative DNA-binding transcription regulators for the presence of HTH motifs. The classification of *C. pseudotuberculosis *FRC41 proteins into Clusters of Orthologous Groups of proteins [111] during genome annotation provided further indications on the role of the predicted proteins in transcriptional regulation. The genome-wide search was extended by using data on known transcription regulators from *C. glutamicum *[23]. A validation step was included by performing BLASTP [112] searches against the NCBI protein database and evaluating results generated with the Conserved Domain Search program [56]. During the final step of data analysis, the DNA-binding transcription regulators of *C. pseudotuberculosis *FRC41 were grouped into regulatory protein families [23,26].

The detection of DNA-binding sites in the genome sequence of *C. pseudotuberculosis *FRC41 followed a combined workflow, using both position weight matrices and hidden Markov models [74]. The programs PoSSuMsearch [113] and HMMsearch [109] were applied to scan the complete genome sequence of *C. pseudotuberculosis *FRC41. As HMMsearch does not support searching in both directions of a double strand, scanning of the reverse complementary DNA sequence was implemented. PoSSuMsearch was configured for lazy probability evaluation [113]. The comprehensive set of validated DNA-binding sites sequences from *C. glutamicum *was downloaded from the reference database CoryneRegNet [114] and used as input for PoSSuMsearch and HMMsearch. The respective gene-regulatory network transfer between *C. glutamicum *and other corynebacteria on the genome-scale was described previously [114]. E-value cut-offs were used as described for the genome-wide pattern recognition approach in *C. glutamicum *[75]. Sequence logos of the detected DNA-binding sites were generated with WebLogo version 2.8.2 [115].

## Authors' contributions

ET sequenced and annotated the FRC41 genome and prepared the manuscript. JSchn implemented and maintained the GenDB project for annotation. JSchr participated in the gene-regulatory network analysis. SJ and AG provided bioinformatic support. PH and JSt participated in the gap closure process. FAD and FSR purified the genomic DNA. LO, SCS, VD, AM, JR and AS participated in data evaluation. NG performed the taxonomic classification of FRC41. OFJL and SK discovered FRC41 and provided the isolate for the sequencing project. VA, AB and AT supervised the project. All authors read and approved the final version of the manuscript.
